# Recall Responses from Brain-Resident Memory CD8^+^ T Cells (bT_RM_) Induce Reactive Gliosis

**DOI:** 10.1016/j.isci.2019.10.005

**Published:** 2019-10-04

**Authors:** Sujata Prasad, Shuxian Hu, Wen S. Sheng, Priyanka Chauhan, James R. Lokensgard

**Affiliations:** 1 Neurovirology Laboratory, Department of Medicine, University of Minnesota, 3-107 Microbiology Research Facility, 689 23^rd^ Avenue S.E., Minneapolis, MN 55455, USA

**Keywords:** Immune System Disorder, Immunology, Neuroscience, Virology

## Abstract

HIV-associated neurocognitive disorders (HAND) persist even during effective combination antiretroviral therapy (cART). Although the cause of HAND is unknown, studies link chronic immune activation, neuroinflammation, and cerebrospinal fluid viral escape to disease progression. In this study, we tested the hypothesis that specific, recall immune responses from brain-resident memory T cells (bT_RM_) could activate glia and induce neurotoxic mediators. To address this question, we developed a heterologous prime-central nervous system (CNS) boost strategy in mice. We observed that the murine brain became populated with long-lived CD8^+^ bT_RM_, some being specific for an immunodominant Gag epitope. Recall stimulation using HIV-1 AI9 peptide administered *in vivo* resulted in microglia displaying elevated levels of major histocompatibility complex class II and programmed death-ligand 1, and demonstrating tissue-wide reactive gliosis. Immunostaining further confirmed this glial activation. Taken together, these results indicate that specific, adaptive recall responses from bT_RM_ can induce reactive gliosis and production of neurotoxic mediators.

## Highlights

• Heterologous prime-CNS boost induced HIV-1-specific bT_RM_, which persisted long term
• Recall responses from HIV-specific bT_RM_ induced tissue-wide reactive gliosis
• bT_RM_ induced-reactive gliosis likely has cumulative neurotoxic consequences

## Graphical Abstract

**Figure:**
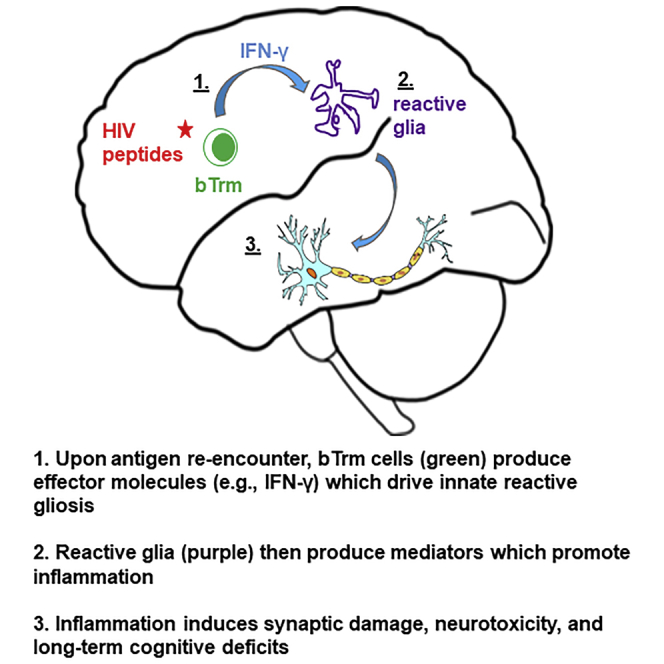

## Introduction

It is currently unknown why HIV-associated neurocognitive disorders (HAND) persist despite effective viral suppression to undetectable levels in most combination antiretroviral therapy (cART)-treated individuals. Although patients on successful cART show sustained viral suppression, as indicated by routine plasma monitoring and occasional cerebrospinal fluid (CSF) monitoring, “blips” indicative of transient HIV replication are often detected with more frequent testing, as reviewed in Chen et al. (2014). A number of studies link persistent immune activation, neuroinflammation, and CSF viral escape in cART-treated individuals to increased disease progression, as well as an increased risk for HAND (Childers et al., 2008, Gisslen et al., 2005, Monteiro de Almeida et al., 2005). Interruption of cART has been associated with CSF viral escape and neuronal injury, as well as with worsened neurocognitive performance (Childers et al., 2008, Gisslen et al., 2005, Monteiro de Almeida et al., 2005). Although fulminant encephalitis is now uncommon in HIV-infected patients, biomarkers of continuing neuroinflammation in the CSF and brain, as well as neuronal injury, are frequently detected in virally suppressed individuals. Still, partial protection against HAND by successful cART does indicate some direct link to HIV replication. Persistent HIV infection and reactivation from central nervous system (CNS) reservoirs, even if intermittent, appears likely in cART-experienced patients (Stam et al., 2013). Brain infection may also be re-seeded through blood or through recently described meningeal lymphatics (Lamers et al., 2016). In addition, evidence for the involvement of CD8^+^ T cells, especially through production of interferon (IFN)-γ, in CNS pathogenesis in patients who received cART continues to mount (Schrier et al., 2015). Therefore, CSF viral escape and the associated production of viral antigen (Ag), as well as its generation of subsequent adaptive, recall responses by brain-resident CD8^+^ T cells to control viral spread, may induce neuroinflammation that drives bystander CNS injury.

Resolution of adaptive immune responses and generation of immunological memory is an essential process to confer long-term protective immunity, particularly in tissues like the brain. The presence of CD8^+^ T cells in post-mortem brains of HIV-infected patients, as well as in the brains of animal models, is well documented (Ganesh et al., 2016, Graham et al., 2011, Grauer et al., 2015, Ho et al., 2013, Kowarik et al., 2014, Marcondes et al., 2007, Schrier et al., 2015). Recent studies have demonstrated that following clearance of many acute viral infections, CD8^+^ T lymphocytes generate a population of long-lived, non-recirculating tissue-resident memory T cells (T_RM_) in non-lymphoid tissue, and it is becoming increasingly clear that these T_RM_ play critical roles in controlling re-encountered pathogens and accelerating the process of clearance (Mackay et al., 2013, Masopust et al., 2010, Park and Kupper, 2015, Schenkel and Masopust, 2014). It is well established that during acute viral infection, most pathogens are rapidly cleared by the generation of a large number of short-lived effector T cells (SLEC), which die via apoptosis once cognate Ag is cleared. Simultaneously, the T cell response is also triggered to generate a defined subset identified as memory precursor effector cells (MPEC). These MPEC begin to develop into a T_RM_ phenotype shortly after infection. Recent work by several groups provides evidence that there is a clear distinction between terminal effector and memory cells based on heterogeneity in expression of killer cell lectin-like receptor G1 (KLRG1) (Bengsch et al., 2007, Kaech and Wherry, 2007, Yuzefpolskiy et al., 2015).

We have recently characterized brain-infiltrating T cells that persist within the CNS after acute murine cytomegalovirus (MCMV) infection (Prasad et al., 2015). We have also shown that these brain CD8^+^ T cell populations shift from SLEC that clear infection to MPEC that protect against viral reactivation and re-challenge. The shift of prominent SLEC populations to MPEC populations is concomitant with transition from acute through chronic phases of infection. T_RM_ are characterized by their non-recirculating, resident nature in tissues. It has been well reported that T_RM_ often express αEβ7 integrin, and integrin αE, otherwise known as CD103, is used as a marker of particular types of T_RM_. High expression of CD103 and CD69 is a common feature of resident memory cells observed in epithelial tissue, as well as a phenotypic signature of bT_RM_ (Steinbach et al., 2016, Wakim et al., 2010, Watanabe et al., 2015, Woon et al., 2016). In contrast, effector and memory cells in circulation appear to lack expression of both CD103 and CD69 (Gebhardt and Mackay, 2012, Masopust et al., 2006). It has also been shown that CD69 expression is required for optimal formation of T_RM_ following herpes simplex virus infection in tissues such as the skin and dorsal root ganglia (Mackay et al., 2013, Mackay et al., 2015b). In addition to CD103^+^ and CD69^+^ markers, T_RM_ are often reported to express CD49a. CD49a constitutes the α subunit of α1β1 integrin receptor, also known as very late Ag 1 (VLA-1) (Cheuk et al., 2017). Experiments using skin, lung, and gut show differential expression of CCR7, as well as CXCR3, which define migration properties of T cells (Mueller and Mackay, 2016, Slutter et al., 2013). Although imperfect, expression of these markers is frequently used to identify T_RM_ populations when stringent migration studies, such as parabiosis, are not feasible, reviewed in Schenkel and Masopust (2014). Finally, protection of the CNS from reinfection by lymphocytic choriomeningitis virus (LCMV) was found to depend on these brain-resident memory CD8^+^ T cells (bT_RM_) (Steinbach et al., 2016).

Given their significance in antiviral defense, surprisingly little is known about this brain-resident memory cell population in the context of HAND. Ag-specific lymphocytes residing within tissues are uniquely poised to respond rapidly to their cognate Ag, and T_RM_ functions extend far beyond cytotoxic T lymphocyte activity. In several infection models, it is clear that recall responses of T_RM_ to small amounts of Ag result in production of IFN-γ (McMaster et al., 2015, Steinbach et al., 2016). Furthermore, this IFN-γ production results in IFN-stimulated gene expression in surrounding cells, thereby amplifying the activation of a small number of adaptive immune cells into an organ-wide antiviral response (Ariotti et al., 2014). Similarly, activation of adaptive immune responses from T_RM_ has been shown to stimulate protective, innate responses in the LCMV model (Schenkel and Masopust, 2014). From these studies, it is clear that a small number of T_RM_ accelerate pathogen control in the event of reinfection or reactivation of latent infection by instructing tissue-resident innate immune cells, such as microglia. Yet there have been no definitive experiments to evaluate neurotoxic consequences of anti-HIV recall immune response by bT_RM_ in driving tissue-wide activation of brain-resident microglial cells, its associated neurotoxicity, and development of subsequent neurocognitive impairment. Here, we demonstrate that HIV-specific T_RM_ are elicited within brain using a heterologous prime-CNS boost strategy. We show that Ag-specific CD8^+^ bT_RM_ are established, which recognize an immunodominant viral epitope. Subsequent recall responses to specific epitope peptides resulted in striking upregulation of major histocompatibility complex (MHC) class II (MHC-II) and programmed death-ligand 1 (PD-L1) on microglial cells, indicative of neuroinflammation. Taken together, data presented here show that recall responses from bT_RM_ can induce reactive gliosis.

## Results

### Ag-Specific CD8^+^ T Cells Infiltrate and Are Retained within the Brain following Priming with rAd5-p24 and CNS Boosting Using Pr55^Gag/Env^ HIV-VLPs

**Figure 1:**
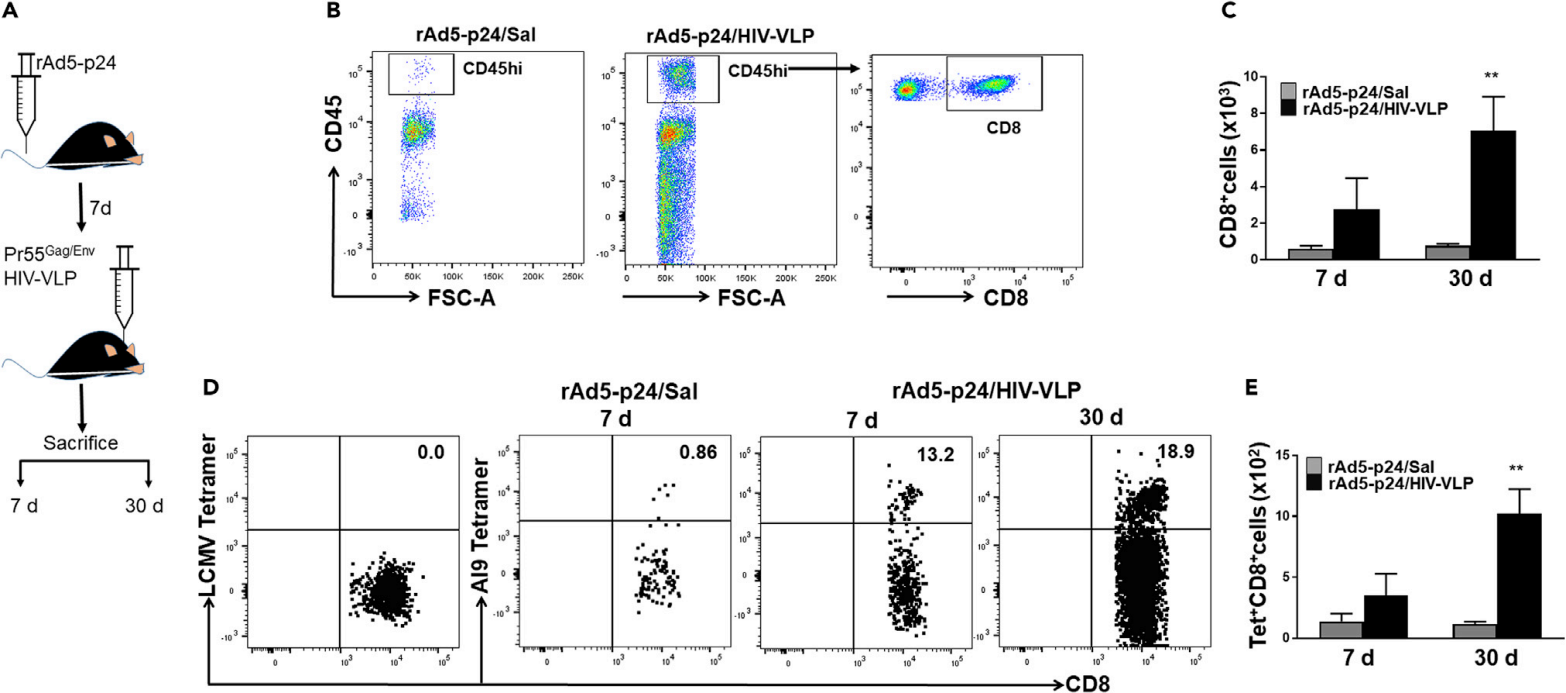
Lymphocyte Infiltration into the Brain and Long-Term Presence of Ag-Specific CD8^+^ T Cells following Heterologous Prime-CNS Boost (A) Schematic of the experimental model illustrates intravenous delivery of recombinant adenovirus vectors expressing HIV-1 p24 capsid protein (rAD5-p24), followed by a CNS-boost consisting of intracranial injection of HIV-VLPs into the striatum. BMNC were collected at 7 and 30 days post prime-boost. (B) Flow cytometric analysis demonstrating lymphocyte infiltration and CD8^+^ T cell retention within the brain following heterologous CNS boost. (C) Absolute numbers of CD8^+^ T cells were determined within brains of animals at the indicated time points. (D) Dot plot comparing the frequencies of AI9 tetramer-specific CD8^+^ T cells in brain tissue isolated from rAd5-p24/Sal (saline) and rAd5-p24/HIV-VLP groups. (E) Bar graph presents absolute numbers of T cells between groups. Pooled data are presented as mean ± SD of two independent experiments using four to six animals per group per time point. **p < 0.01

To investigate whether recall responses from bT_RM_ can induce reactive gliosis, we first established a population of HIV-specific T cells within the murine brain using a heterologous prime-CNS boost strategy. To accomplish this, BALB/c mice were primed via tail vein injection with recombinant adenovirus vectors expressing the HIV-1 p24 capsid protein (rAd5-p24). This priming step was followed by a CNS-boost using Pr55^Gag/Env^ virus-like particles (HIV-VLPs) injected directly into the striatum (Figure 1A). Using immunohistochemical (IHC) staining, we first confirmed that the adenovirus construct delivered bona fide HIV-p24 protein by staining murine liver sections 7 days post-priming (Figure S1 A). We then detected increased lymphocyte trafficking into the brains of rAd5-p24-primed animals, which received an HIV-VLP CNS boost when compared with control animals that were primed, but did not receive HIV-1 Ag in the brain (Figures 1B and 1C). We observed that CD8^+^ T cells infiltrated the brain and were present at 7 days post HIV-VLP injection, and further increased at 30 days post prime-CNS boost (Figure 1C). In addition, we used tetramer staining to confirm that AI9-specific, but not LCMV epitope-specific, CD8^+^ T cells were present at 7 days and continued to persist until at least 30 days post prime-CNS boost (Figures 1D and 1E). We also examined the recruitment of CD4^+^ T cells and observed an influx into the brain at 7 days following prime-CNS boost (38.3%). The frequency of CD4^+^ T cells increased by 30 days (42.2%), similar to that observed for CD8^+^ T cells. However, by 60 days the frequencies of both CD8^+^ and CD4^+^ T cells decreased (20.8% and 32.9%, respectively). In addition, as a result of AI9 *in vivo* restimulation, the frequency of CD8^+^ T cells was elevated by 2 days post-peptide injection, whereas CD4^+^ T cell frequency was not (Figure S2A).

### Memory CD8^+^ T Cells Are Present within the Brain following Prime-CNS Boost Strategies

**Figure 2:**
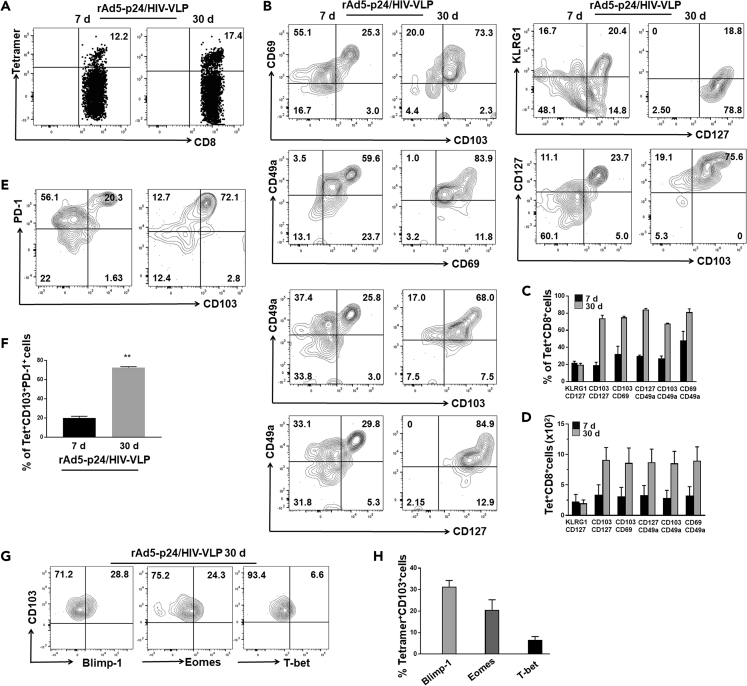
Phenotypic Characterization of Ag-Specific T_RM_ within the Brain (A and B) CNS-derived lymphocytes were gated for AI9-specific CD8^+^ T cells and analyzed for the indicated memory cell markers (CD127, CD103, CD69, CD49a), as well as the short-lived effector marker KLRG1. (C and D) Bar graphs present pooled frequencies and number of Ag-specific cells that expressed the indicated phenotypic markers. (E) Additional contour plots show PD-1 expression on these Ag-specific CD103^+^ CD8^+^ T cells at 7 and 30 days post prime-boost. (F) Pooled data show frequency of PD-1 expression on Ag-specific CD103^+^CD8^+^ T cells at the indicated time points. **p < 0.01. (G) Representative plots show expression of transcription factors Blimp-1, Eomes, and T-bet at the indicated time point. (H) Pooled data present percentage of Blimp-1, Eomes, and T-bet expression. Graph presents data combined from two separate experiments using four to six animals per group per time point.

To characterize HIV-specific CD8^+^ T cells within the brain, we analyzed Ag-specific cells for phenotypic markers at both 7 and 30 days post prime-CNS boost (Figure 2A). We first determined the pattern of CD127 and KLRG1 expression on these Ag-specific cells. We observed expression of CD127 on some Ag-specific CD8^+^ T cells during the acute phase of infection, whereas a larger fraction of AI9 tetramer^+^ CD8^+^ T cells displayed high CD127 expression at 30 days post prime-CNS boost (Figure 2B). In sharp contrast, KLRG1 expression was found to be 37.8% ± 3.4% among Ag-specific cells during onset of infection, whereas it decreased to 19.8% ± 2.7% at 30 days (Figures 2B and 2C). To gain insight into the diversity of T_RM_ phenotypes, we next examined the relative expression of various residency markers such as CD103, CD69, and CD49a. An increased expression of CD69 at 7 days suggested that these Ag-specific CD8^+^ T cells progress to an activated state. Expression of CD49a was also observed during establishment of infection. Interestingly, expression of both CD69 and CD49a remained elevated even at 30 days. Their increased co-expression was also observed during the chronic phase (81.1% ± 3.8%; Figures 2B and 2C). Moreover, high co-expression of CD69 and CD103 (74% ± 3.5%), as well as CD127 and CD103 (75% ± 1.3%), was observed. In addition, an increased proportion of Ag-specific CD8^+^ T cells was found to co-express CD49a and CD103 (67.9% ± 0.3%), as well as CD49a and CD127 (84.2% ± 1.1%) (Figures 2B and 2C). Evaluation of various memory marker-expressing cells revealed greater numbers of T_RM_ at 30 days than at 7 days following prime-CNS boost (Figure 2D). Finally, we also observed that these Ag-specific CD8^+^CD103^+^ T cells also expressed programmed cell death (PD)-1 (72.5% ± 0.8%) (Figures 2E and 2F). Furthermore, evaluation of additional transcription factors associated with tissue residency revealed elevated levels of B lymphocyte-induced maturation protein (Blimp)-1 and eomesodermin (Eomes), whereas reduced levels of T-box expressed in T cells (T-bet) were seen at 30 days post prime-CNS boost (Figures 2G and 2H).

### Effector Responses of bT_RM_

**Figure 3:**
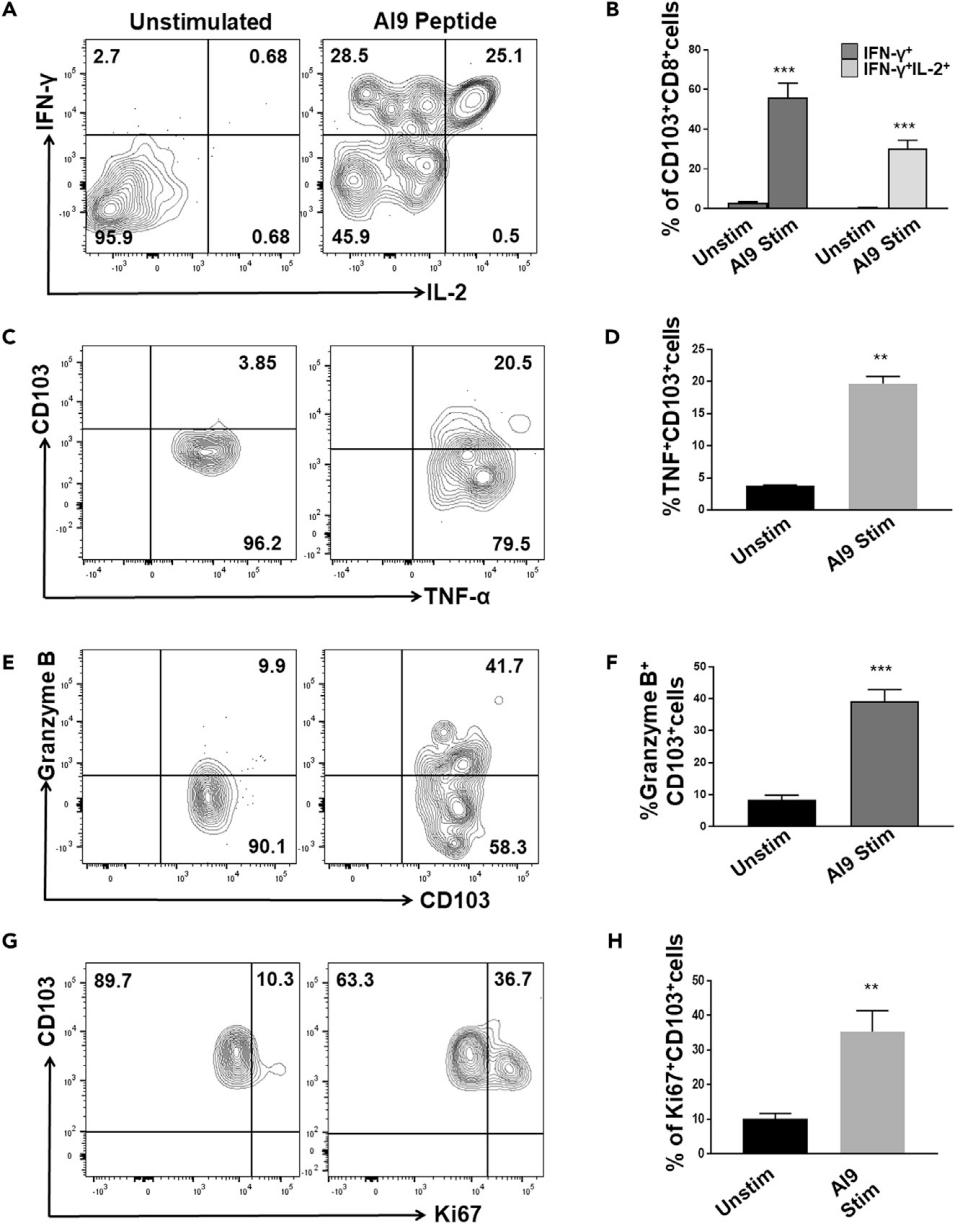
Ag-Specific CD103^+^CD8^+^ T Cells Display Recall Responses BMNC obtained from animals at 30 days post prime-CNS boost were either restimulated *ex vivo* with AI9 peptide or left unstimulated, and intracellular staining was performed. Gated CD103^+^CD8^+^ T cells were assessed for cytokine production in response to peptide stimulation. (A) Representative contour plots present expression of IFN-γ and IL-2 by CD103^+^CD8^+^ T cells. (B) Bar graph of pooled data shows frequencies of IFN-γ production by CD103^+^CD8^+^ T cells, as well as simultaneous detection of IFN-γ and IL-2 double-producer cells. (C) Contour plots show TNF-α production by CD103^+^CD8^+^ T cells. (D) Pooled data show the frequency of TNF-α production. (E) Representative contour plots of granzyme B show *ex vivo* production of this cytotoxic mediator in response to peptide stimulation. (F) Data presented show percentage of granzyme B-producing CD103^+^CD8^+^ T cells. (G) Contour plots following Ki67 staining show proliferation frequencies of CD103^+^CD8^+^ T cells, either with or without peptide stimulation. (H) Pooled data present percentage proliferation of bT_RM_. **p < 0.01 and ***p < 0.001

To investigate the ability of bT_RM_ to respond to restimulation, we evaluated the production of IFN-γ and interleukin-2 (IL-2), as well as granzyme B. Analysis was performed using intracellular staining and flow cytometry following *ex vivo* stimulation with AI9 peptide, which is an immunodominant, H-2K^d^ MHC-I-restricted T cell epitope from the p24 (65-73) capsid. Brain-derived CD8^+^CD103^+^ T_RM_ produced IFN-γ following peptide stimulation. Higher frequencies (55.8% ± 7.3%) were produced following *ex vivo* peptide stimulation when compared with unstimulated control (3.0% ± 0.43%, p < 0.05) (Figures 3A and 3B). We also analyzed dual cytokine production by assessing IFN-γ and IL-2 co-expression. A substantial percentage of dual IFN-γ- and IL-2-producing CD8^+^CD103^+^ T cells (30.4% ± 4.0%) was detected following peptide restimulation (Figures 3A and 3B). Increased production of tumor necrosis factor (TNF)-α was also noted in response to *ex vivo* AI9 restimulation (Figures 3C and 3D). In addition, granzyme B-expressing cells were also detected in a larger proportion (39.2% ± 3.6%) when compared with unstimulated control (8.4% ± 1.3%) (Figures 3E and 3F). Finally, we analyzed the proliferation potential of these CD103^+^CD8^+^ T cells following Ag stimulation and found that Ki67 was expressed in a significant fraction, whereas unstimulated cells displayed lower levels (35.3% ± 6.1% and 10.2% ± 1.4%, respectively) (Figures 3G and 3H).

### Glial Cell Activation following Peptide Restimulation

**Figure 4:**
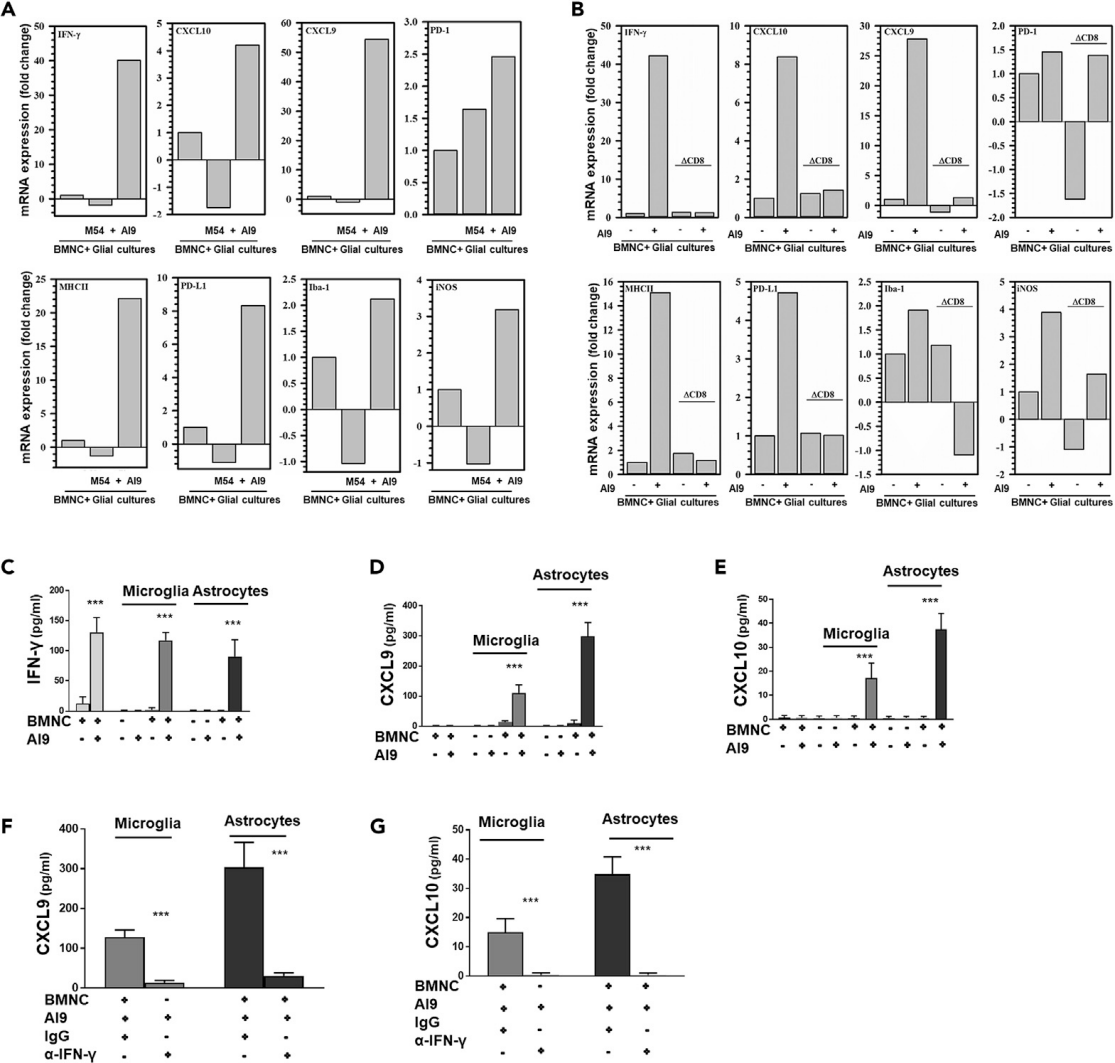
Specific *Ex Vivo* Peptide Restimulation of T Cells Activates Primary Glial Cells (A) BMNC isolated from brains of 30 days post prime-boost animals were co-cultured with primary murine glial cells (40% microglia; 60% astrocytes). Cells were either treated with HIV-specific AI9 or MCMV M54 T cell epitope peptides or left untreated for 24 h. Real-time PCR data show mRNA expression levels of IFN-γ, MHC-II, PD-L1, PD-1, Iba-1, CXCL9, CXCL10, and nitric oxide synthase (iNOS) under the indicated treatment. (B–G) (B) Cultures with or without the CD8 T cell population were either stimulated with AI9 peptide or left unstimulated for 1 h before being co-cultured with mixed glial cells (40% microglia; 60% astrocytes) for 24 h. qPCR data presenting transcript levels of IFN-γ, MHC-II, PD-L1, PD-1, Iba-1, CXCL9, CXCL10, and iNOS under the indicated treatment. Data shown are representative of two separate experiments. Supernatants from AI9-stimulated or unstimulated BMNC co-cultured either with microglial cells or astrocytes were collected at 48 h and ELISA was performed for IFN- γ (C), CXCL9 (D), and CXCL10 (E). Blocking of IFN-γ using a neutralizing anti-IFN-γ antibody inhibited CXCL9 (F) and CXCL10 (G) expression. The results shown are from pooled data of two independent experiments. ***p < 0.001.

Having observed that *ex vivo* stimulation resulted in cytokine production, we went on to determine the capacity of these viral-peptide-induced recall responses to activate glia. In these experiments, brain-derived mononuclear cells (BMNC) obtained from prime-CNS boost mice at 30 days were co-cultured with primary murine glial cells (40% microglia, 60% astrocytes) and stimulated with either the AI9 peptide or the non-specific M54 peptide epitope from MCMV. Increased transcription of proinflammatory mediators (i.e., IFN-γ, CXCL9, CXCL10, nitric oxide synthase [iNOS], and PD-1) was observed in the co-cultures after 24 h of stimulation with specific (AI9), but not non-specific (M54), T cell epitope peptides (Figure 4A). Also, elevated levels of MHC-II, PD-L1, and Iba (ionized calcium-binding adaptor molecule)-1 mRNA indicated glial cell activation in response to AI9, but not M54, peptide (Figure 4A). To specifically evaluate the role of CD8^+^ T cells in modulating glial activation, we used positive selection to deplete this T cell population from the BMNC isolated from prime-CNS boost animals at 30 days. Depletion of CD8^+^ T cells was confirmed using flow cytometry (Figure S2B). The sorted BMNC (i.e., with and without CD8^+^ T cells) were then cultured with mixed glial cells, and transcription of the same proinflammatory mediators was assessed after 48 h. Depletion of CD8^+^ T cells was found to reduce transcription of IFN-γ as well as the IFN-γ-induced chemokines CXCL9 and CXCL10. Reductions in transcription of MHC-II, PD-L1, Iba-1, iNOS, and PD-1 were also observed in the absence of CD8^+^ T cells (Figure 4B). Also, to determine the individual contribution of each glial cell type, we assessed cytokine and chemokine production at the protein level from co-cultures of AI9-stimulated BMNC reconstituted with either primary microglial cells or primary astrocytes. ELISA results indicated that BMNC produced IFN-γ, in response to AI9 restimulation, either when cultured alone or when reconstituted with either microglia or astrocytes (Figure 4C). Elevated levels of CXCL9 (Figure 4D) and CXCL10 (Figure 4E) were found in the BMNC-glial cell co-cultures, but not from AI9-stimulated BMNC alone. Co-cultures of unstimulated BMNC (i.e., no AI9 treatment) with either microglia or astrocytes produced negligible levels of IFN-γ, CXCL9, or CXCL10 (Figure 4C–4E). Interestingly, treatment of the co-cultures with an anti-IFN-γ neutralizing antibody significantly reduced subsequent glial cell expression of CXCL9 and CXCL10 when compared with IgG isotype antibody (Figures 4F and 4G).

### *In vivo* Recall Responses Stimulate Rapid Proliferation of HIV-Specific bT_RM_ within the Brain

**Figure 5:**
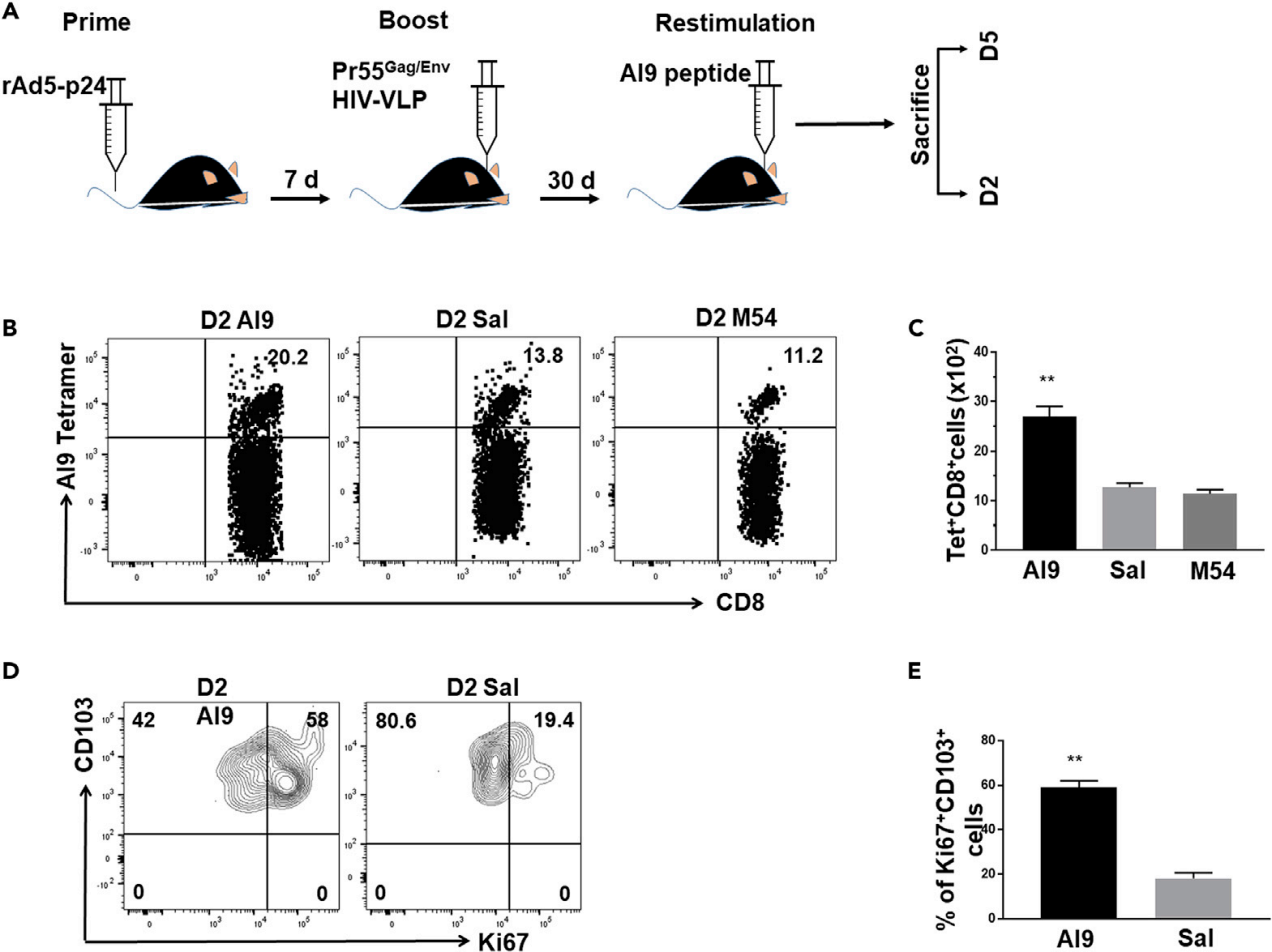
*In Vivo* Recall Responses Stimulate Proliferation of Brain-Resident HIV-Specific CD8^+^ T Cells BMNC obtained from prime-CNS boost animals were collected and evaluated using flow cytometry at the indicated time points following *in vivo* peptide restimulation. (A) Schematic representation of experimental approach to evaluate *in vivo* recall responses. The AI9 peptide, an irrelevant T cell epitope peptide (M54), or saline were injected into the brain and tissues were collected at 2 and 5 days post-restimulation. (B) Dot plots show tetramer-specific CD8^+^ T cells in the brain at 2 days post-peptide restimulation. (C) Bar graph presents numbers of HIV-specific CD8^+^ T cells following the indicated restimulation. (D) Contour plots present Ki67 expression on CD103^+^CD8^+^ T cells at 2 days post-restimulation with cognate peptide. (E) Bar graph represents the pooled frequencies of Ki67-expressing CD103^+^CD8^+^ T cells. Data are presented as mean ± SD of two independent experiments using four animals in control or irrelevant peptide groups and six animals following AI9 restimulation. **p < 0.01

It is well-established that T_RM_ respond rapidly to an Ag re-challenge (Schenkel and Masopust, 2014). Our *ex vivo* studies demonstrated that IFN-γ and IFN-γ-inducible chemokines, as well as microglial activation makers, were produced following peptide-specific stimulation. To study recall responses *in vivo*, we intracranially administered either the Ag-specific peptide AI9 or an irrelevant peptide or saline to animals at 30 days post prime-CNS boost (Figure 5A). We first found that recall activation following injection of HIV-specific peptide resulted in an increased number of AI9 tetramer^+^ CD8^+^ T cells within the brain (Figure 5B). This effect was specific to AI9 peptide and was not observed with irrelevant peptide (M54) or saline (Sal) (Figures 5B and 5C). We went on to determine that brain-resident CD103^+^CD8^+^ T cells expanded following *in vivo* restimulation. Ki67 staining demonstrated this increased bT_RM_ proliferation in response to AI9 restimulation relative to the control group (Figures 5D and 5E).

### Recall Responses Induce Glial Cell Activation

**Figure 6:**
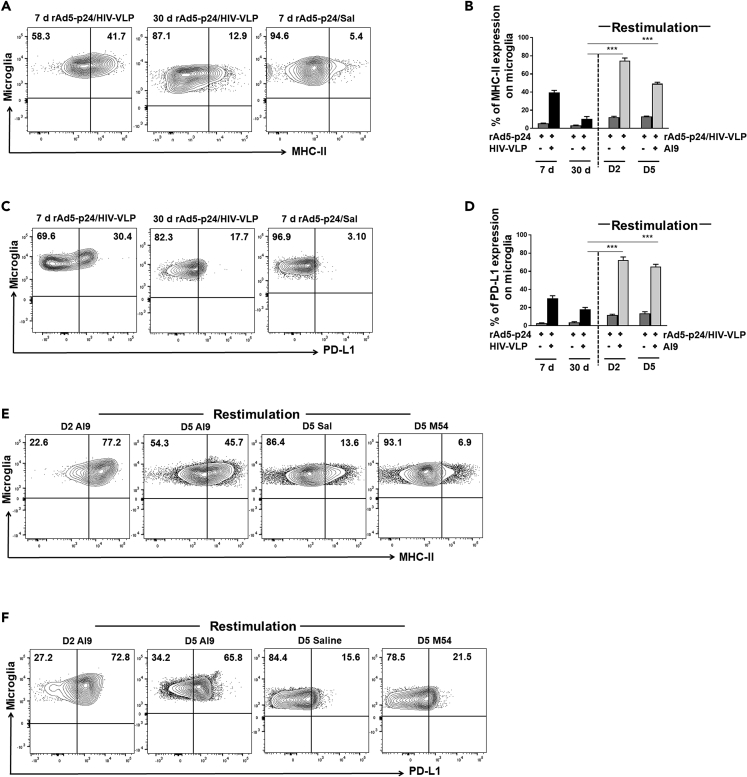
*In Vivo* Recall Responses by bT_RM_ Induce Microglial Cell Activation Flow cytometry and IHC staining were performed on brain tissue obtained from mice following prime-CNS boost. (A) Contour plots present expression of MHC-II on CD45^int^/CD11b^+^ microglia at 7 and 30 days post prime-CNS boost. (B) Pooled data show the frequencies of MHC-II expression at the indicated time points both pre- and post-recall restimulation via intracranial injection of AI9 peptide. (C) Contour plots present expression of PD-L1 on stimulated CD45^int^/CD11b^+^ microglial cells at 7 and 30 days post prime-boost. (D) Bar graph presented shows frequencies of PD-L1 expression at the indicated time points both pre- and post-restimulation. (E) Representative contour plots show percentage of microglia expressing MHC-II at 2 and 5 days post *in vivo* AI9 restimulation. (F) Representative contour plots show the percentage of microglial cells expressing PD-L1 at 2 and 5 days post *in vivo* restimulation. Data presented are mean ± SD of two independent experiments using six animals in control and irrelevant peptide groups at all time points and six to eight animals in the AI9 restimulation group at 7- and 30-day time points, respectively. ***p < 0.001.

Having observed that T_RM_ isolated from the brain produce abundant proinflammatory cytokines following AI9 peptide stimulation *ex vivo*, we next investigated the *in vivo* induction of reactive gliosis. We first assessed the kinetics of MHC-II and PD-L1, which are markers of activation, expression on microglial cells at both 7 and 30 days post prime-CNS boost. Unstimulated microglia, identified as the CD45^int^CD11b^+^ population using flow cytometry (Figure S3A), have been reported to express minimal levels of MHC-II and PD-L1 (Figures S3B and S3C), whereas a reactive microglial cell phenotype was observed following our prime-CNS boost regimen. Increased expression of MHC-II on microglial cells was first observed during the acute phase (39.5% ± 2.4%) and remained elevated at 30 days post prime-CNS boost (10.5% ± 2.5%), when compared with the control group 5.3% ± 0.4% and 3.2% ± 0.4% at 7 and 30 days, respectively (Figures 6A and 6B). Similarly, expression of PD-L1 was also upregulated at 7 days (30.3% ± 2.6%) and it continued to be expressed at the 30-day time point (17.9% ± 2.2%) (Figures 6C and 6D). PD-L1 expression on microglia was minimal in the control animals (2.8% ± 0.3% and 3.7% ± 0.7% at 7 and 30 days, respectively).

To investigate the effect of recall responses on brain-resident glia, we examined microglial cell activation at 2 and 5 days following restimulation via intracranial injection of AI9 peptide (i.e., at 30 days post prime-CNS boost). We again analyzed MHC-II and PD-L1 expression as microglial activation markers. Interestingly, specific Ag restimulation rapidly induced upregulation of MHC-II expression on microglia cells (74.6% ± 2.9% at 2 days and 49.2% ± 1.6% at 5 days post-restimulation) (Figures 6E and 6B). Similarly, microglia also displayed an increase in PD-L1 (72.4% ± 3.3% at 2 days) that remained elevated (65.1% ± 2.6%) at 5 days following AI9 peptide. Importantly, glial cell activation was not observed in response to MCMV M54 peptide restimulation or saline (Figures 6F and 6D). In addition, i*n vivo* recall immune responses from bT_RM_ may also augment cytotoxic activity within the brain (Figures S4A and S4B).

### Reactive Glial Cell Phenotypes in Response to *In Vivo* AI9 Restimulation

**Figure 7:**
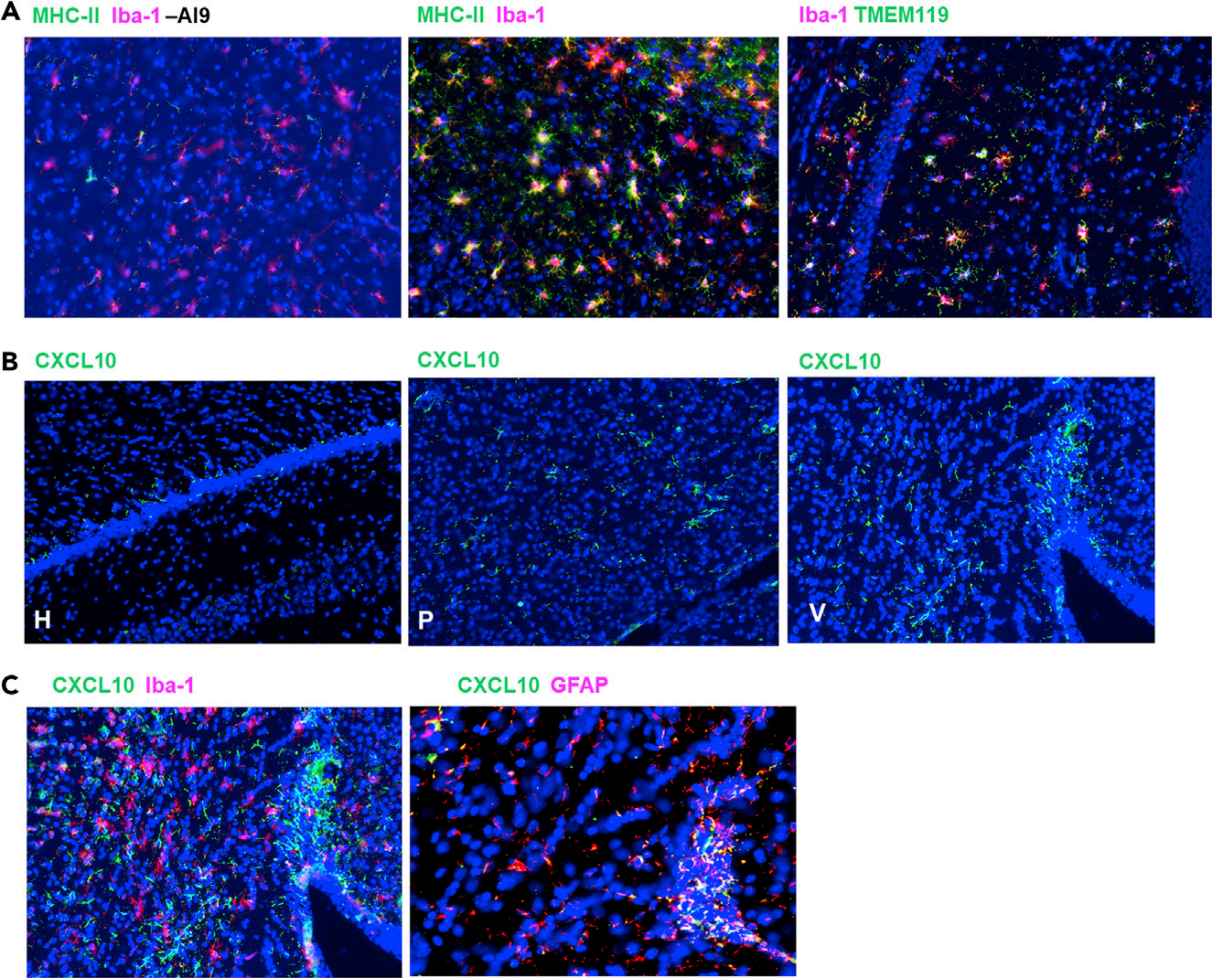
Recall Responses from bTRM Cells Induce Reactive Glial Cell Phenotypes (A) Dual IHC staining of brain sections obtained from animals at 2 days post-AI9 restimulation for MHC-II and Iba-1, as well as Iba-1 and TMEM119. (B) IHC staining displaying expression of CXCL10 in the hippocampus (H), parenchyma (P), and ventricle (V) of AI9-restimulated animals. (C) Images of CXCL10 co-staining with Iba-1, as well as co-expression of CXCL10 and GFAP.

We performed IHC on sections of brain obtained from primed-CNS boost mice following 2-day recall stimulation (+AI9), co-staining for MHC-II and Iba-1. Microglial cells within these sections displayed a reactive phenotype, as observed by elevated expression of MHC-II and Iba-1 (Figure 7A). Double immunostaining with anti-TMEM119 (a microglial cell-specific marker) and Iba-1 revealed co-localization following *in vivo* AI9 restimulation (Figure 7A). Expression of CXCL10 was also seen in several locations within the restimulated brain: it was evident in the hippocampus, ventricles, and parenchyma (Figure 7B). Most interestingly, dual staining of CXCL10 with Iba-1, as well as the astrocyte marker glial fibrillary acidic protein (GFAP), demonstrated greater co-localization of CXCL10 in the GFAP^+^ population than in the Iba-1^+^ cells (Figure 7C). This finding demonstrated that specific recall responses of bT_RM_ also stimulated astrocytes.

## Discussion

During early HIV-1 infection, CD8^+^ T cell responses play essential roles in viral clearance. As infection proceeds, T_RM_ develop and persist long term in a number of tissues, such as the gastrointestinal tract and female reproductive tract (Kiniry et al., 2018, Tan et al., 2018). These T_RM_ provide robust and immediate effector immune responses to localized reinfection or reactivation of Ag production. The brain is recognized as an important reservoir for latent HIV-1, as well as viral reactivation (Hellmuth et al., 2015). Like other tissues, T_RM_ formation within brain has been demonstrated in several viral infection models (Gebhardt and Mackay, 2012, Prasad et al., 2017, Wakim et al., 2010). It is particularly important to understand recall responses of bT_RM_ because although they appear to be critical in controlling virus, long-term persistent neuroinflammation may lead to deleterious neurocognitive consequences.

Prime-boost regimens have emerged as powerful tools to enhance tissue-infiltrating Ag-specific T cells (Tan et al., 2016, Tan et al., 2018, Zaric et al., 2017). Likewise, the heterologous prime-CNS boost approach used in this study was able to induce brain infiltration of Ag-specific CD8^+^ T cells. A fraction of these bT_RM_ can be isolated from brain tissue using *ex vivo* dissociation methods. It has been reported that lymphocyte extraction methods fail to efficiently capture all the cells from non-lymphoid tissue and that the efficiency of T_RM_ isolation varies greatly between histologically distinct locations (Steinert et al., 2015). However, the heterologous prime-CNS boost approach used here does provide for the long-term retention of Ag-specific memory cells within the CNS. This observation is in agreement with studies by others wherein Ag-specific CD8^+^ T cells are preferentially retained in non-lymphoid tissue, even after resolution of infection (Khan et al., 2016, Tan et al., 2016, Tan et al., 2018).

Results presented here demonstrate that long-term retention of HIV-specific CD8^+^ T cells within brain is coupled with the presence of markers of T_RM_. High frequencies of CD8^+^CD103^+^CD69^+^CD49a^+^ were apparent on T cells during the chronic phase following prime-CNS boost. Phenotyping of Ag-specific CD8^+^ T cells infiltrating the brain revealed distinct populations of SLEC and MPEC from acute into chronic phases. The KLRG1^+^ population (i.e., SLEC) dominates at 7 days post prime-CNS boost. In contrast, later time points correlate with development of KLRG1^−^ CD127^+^ cells (MPEC). The Ag-specific CD8^+^ T cells expressed surface CD103, an integrin binding to E-cadherin, and co-expressed CD69 at 30 days. Consistent with other studies, where T_RMs_ in barrier tissues express CD49a and display cytotoxic potential (Cheuk et al., 2017), we found that CD49a was expressed along with other markers of tissue residency; specific peptide stimulation induced granzyme B production (Figures S4A and S4B).

In addition to CD103, CD8^+^ T_RM_ in non-lymphoid tissue, including human brain, are defined by the CD69 marker (Khan et al., 2016, Smolders et al., 2018). CD69 is an activation marker expressed early that aids in T_RM_ retention by downregulating cell surface expression of S1P1, thereby blocking T cell movement out of tissues (Mackay et al., 2015a, Park and Kupper, 2015). Our findings are similar to those of other studies, where HIV-specific CD8^+^ T_RM_ were established in murine vaginal mucosa using combined intranasal and intravaginal immunization with recombinant influenza vector (Tan et al., 2018). Generation of long-lived Ag-specific T cell memory has also been demonstrated in mucosal tissue following micro-needle array delivery of adenovirus type 5 vectors encoding HIV-1 Gag (Zaric et al., 2017). Here, we showed that in our prime-CNS boost model, HIV-1-specific CD8^+^ T cells displayed phenotypic signatures indicative of T_RM_. Recent studies identify the transcription factors Blimp-1 and homolog of Blimp-1 in T cells (Hobit) in promoting T_RM_ cell development. Blimp and Hobit cooperate to repress genes such as Klf2, S1pr1, and CCR7 (required for tissue egress) and thus contribute to the establishment of tissue residency (Bird, 2016). Similar to these findings, our study demonstrated expression of Blimp-1, along with low levels of T-bet, indicative of long-lived memory cells. Differential expression of these transcription factors in different microenvironments corresponding to varying degrees of inflammation is likely.

T_RM_ survey for their cognate Ag and initiate tissue-wide inflammation to enhance pathogen clearance. T_RM_ that produce abundant IFN-γ and TNF-α, as well as mediators of cytotoxicity, have been identified in a number of infection models (Muruganandah et al., 2018, Prasad et al., 2015, Steinbach et al., 2016). HIV-1-specific CD8^+^ T_RM_ have been shown to participate in local antiviral immunity (Kiniry et al., 2018). IFN-γ, together with other proinflammatory cytokines, has been shown to induce chemokines and establish chronic immune activation during HIV-1 infection (Roff et al., 2014). In skin, it has been demonstrated that CD49a^+^ T_RM_ produce IFN-γ and rapidly gain cytotoxic capacity in response to IL-15 (Cheuk et al., 2017). Here, we also demonstrated elevated levels of cytokine production and cytolytic potential of T_RM_ in terms of IFN-γ, IL-2, TNF-α, and granzyme B production. IFN-γ promotes long-term microglial cell activation and TNF- α production (Mutnal et al., 2011). The synergistic action of IFN-γ and TNF- α within the brain has been reported to induce nitric oxide-induced neurodegeneration (Blais and Rivest, 2004). *In vivo* recall immune responses from bT_RM_ may also augment cytotoxic activity within the brain. These findings indicate that potent anti-HIV responses develop upon restimulation with specific T cell epitopes. Our findings are similar to those of other studies wherein robust polyfunctional Gag-specific responses of T_RM_ contribute to HIV-1-specific immunity (Tan et al., 2016, Tan et al., 2018, Zaric et al., 2017).

Although the presence of Ag contributes to bT_RM_ proliferation, the local brain microenvironment also modulates their density and influences their survival. bT_RM_ exhibit limited survival and proliferative capacity *in vitro* (Wakim et al., 2010); however, they do possess self-renewing capacity *in vivo* and display high proliferation during recall responses (Steinbach et al., 2016). Similarly, we found increased proliferation of bT_RM_ upon Ag re-challenge, which suggests retention of their proliferative potential during recall responses. It has also been demonstrated that the density of skin T_RM_ influences local immune protection (Cheuk et al., 2017).

We have previously reported that PD-1 is expressed at high levels on bT_RM_ (Prasad et al., 2017). Yet, despite this upregulation, in the current study bT_RM_ still displayed heightened proliferation and increased cytokine production in response to cognate Ag. This observation further supports the hypothesis that PD-1 is not simply a marker of exhaustion. These findings are in line with those of other studies wherein PD-1^+^CD8^+^ T_RM_ displayed transcriptional profiles with effector phenotypes, and showed clonal expansion (Petrelli et al., 2018). PD-1 expression on activated cells is well known to shut down responses to prevent immunopathology (Sharpe et al., 2007). However, in HIV-1 infection it appears that PD-1 expression on bT_RM_ may influence their functional properties in an inflamed brain microenvironment. Therefore, it will be of great interest to further define the roles of PD-1 on bT_RM_ in cART-treated and untreated patients.

T_RM_ are a unique type of memory T cell, specifically tailored to survive in non-lymphoid tissues. However, prolonged bT_RM_-driven recall responses in a microenvironment that normally lacks routine immune surveillance could be problematic. Persistent neuroimmune activation in response to viral reactivation may result in extensive damage to this largely non-regenerating tissue. Published reports show that a low copy number of HIV-1 provirus can still be intermittently detected in the CSF of patients receiving cART (Spudich et al., 2006, Yilmaz et al., 2006), and persistent CNS inflammation is important to consider during HAND therapy (Saylor et al., 2016). In experimental models, despite low levels of virus, chronically HIV-infected NSG-hCD34^+^ mice show features of HAND (i.e., brain pathology including activation of microglia, upregulation of neuroinflammatory markers such as Mac1, an indicator of microgliosis, and iNOS), as well as neurodegeneration (Boska et al., 2014, Saylor et al., 2016). In our study, Ag-specific AI9 restimulation *in vivo* showed rapid upregulation of the microglial activation markers MHC-II and PD-L1 at both 2 and 5 days post-restimulation. Furthermore, real-time RT-PCR data from *ex vivo* restimulation experiments demonstrate that recall responses from bT_RM_ are capable of driving microglial cell activation, chemokine production, and production of iNOS. These data demonstrate that glial cells develop reactive phenotypes following the recall response of bT_RM_ to re-challenge with cognate Ag, but not in response to stimulation with non-specific T cell epitopes. Previous studies from our laboratory have identified glial cells as an important source of proinflammatory cytokines and chemokines (Cheeran et al., 2001, Cheeran et al., 2003, Lokensgard et al., 2016). Reports have shown that the potent T cell chemoattractants CXCL9 and CXCL10 are produced within inflamed brain and that their expression is enhanced by IFN-γ (Lokensgard et al., 2015, Michlmayr and Lim, 2014, Mutnal et al., 2011). Similarly, in this study elevated levels of CXCL9 and CXC10 were observed, expression of which was significantly reduced following blockade of IFN-γ. Production of IFN-γ was found to be specific to the CD8^+^ T cells as demonstrated by our qPCR data. Following *ex vivo* AI9 restimulation, our data indicate that this cytokine is a driving force for glial cell activation. These findings have implications for neurodegenerative processes by releasing cytotoxic factors such as nitric oxide (NO), as well as several neurotoxic proinflammatory cytokines (Chao et al., 1992, Matsuoka et al., 1999).

Properly regulated neuroimmune responses strike a balance between pathogen clearance and an acceptable level of bystander tissue damage. In cART-treated patients with HAND, there is evidence of lingering, long-term astrocyte activation (Vanzani et al., 2006, Woods et al., 2010). As is the case with microglia, astrocytes also respond to T cell- and microglial cell-produced immune mediators to acquire reactive phenotypes (Liddelow et al., 2017). This cellular activation is routinely visualized by an upregulation of GFAP, a marker of astrogliosis (Burda and Sofroniew, 2014). Here, IHC staining of brain sections from prime-CNS boost mice subjected to recall stimulation further demonstrates the effect of bT_RM_ on glial cell reactivity. Co-localization of MHC-II and Iba-1, as well as GFAP and CXCL10, highlights recall-response-driven reactive phenotypes in both these glial cell types.

In summary, the results reported here demonstrate that our heterologous prime-CNS boost model induces a population of HIV-1-specific bT_RM_, which persist long term. As viral reactivation from CNS reservoirs appears likely even in cART-experienced patients, this model was used to demonstrate that antiviral recall responses from bT_RM_ can induce tissue-wide reactive gliosis through cytokine-induced activation of brain-resident glia. This cytokine-induced reactive gliosis may protect against viral spread throughout the brain, but it likely has cumulative neurotoxic consequences.

### Limitations of the Study

There is no ideal animal model that allows for the invasive study necessary to understand HIV reactivation from brain reservoirs and its association with HAND. It is also unlikely that *in vivo* restimulation with AI9 peptide exactly mimics transient viral protein production within human brain. However, use of this heterologous prime-boost model results in the murine brain being populated by resident memory CD8^+^ T cells specific for an immunodominant HIV Gag epitope. Subsequent *in vivo* recall neuroimmune responses can then be induced.

## Methods

All methods can be found in the accompanying Transparent Methods supplemental file.

## Supplemental Information

Document S1. Transparent Methods and Figures S1–S4
