# Supplementary material for: Recall Responses from Brain-Resident Memory CD8+ T Cells (bTRM) Induce Reactive Gliosis
Source: iScience. 2019 Oct 4;20:512–26. doi: 10.1016/j.isci.2019.10.005 (PMC6807101; doi:10.1016/j.isci.2019.10.005)

ISCI, Volume *20*

Supplemental Information

Recall Responses from Brain-Resident Memory CD8^{+} T Cells (bT$_{RM}$) Induce Reactive Gliosis

Sujata Prasad, Shuxian Hu, Wen S. Sheng, Priyanka Chauhan, and James R. Lokensgard

Transparent Methods

METHODS

Ethical statement

This study was carried out in strict accordance with recommendations in the Guide for the Care and Use of Laboratory Animals of the National Institutes of Health. The protocol was approved by the Institutional Animal Care and Use Committee (Protocol Number: 1701-34513A) of the University of Minnesota. All surgery was performed under Ketamine/Xylazine anesthesia and all efforts were made to minimize suffering.

Virus and animals

Six to eight week old BALB/c mice were vaccinated via tail-vein injection with an adenovirus vector (1 x 10^{10} PFU/mouse) expressing the HIV-1 p24 capsid protein (rAd5-p24). We outsourced production of a 2nd-generation (i.e., ΔE1 + ΔE3), replication incompetent adenovirus vector to Cyagen Biosciences (Santa Clara, CA), which expresses the HIV-1 capsid protein p24 under control of the minimal CMV IE promoter (i.e., rAd5-p24). Following vaccination, to promote immune cell infiltration and retention in the brain, animals were then boosted (7d later) via an intracranial injection of HIV virus-like particles (HIV-VLPs). Animals received 300 fluorescent units (FU) of HIVLP in a volume no greater than 5ul delivered to the brain striatum via stereotaxic injection.

Production of HIV virus-like particles (HIV-VLPs)

HIV-VLPs were produced by transfection of HEK 293T cells with pEYFP-N3 HIV-1, a Gag-expressing codon-optimized plasmid encoding the 55kDa Gag precursor protein fused to enhanced yellow fluorescent protein (EYFP), under control of the human CMV IE promoter, obtained from Louis Mansky (Institute for Molecular Virology, University of Minnesota); p3NL(ADA)env,

encoding a full length R5-tropic envelope protein, under control of the HIV-1 LTR promoter, was obtained with permission from Eric Freed (NCI, Frederick, MD). To produce Gag-alone HIV-VLPs, cells are transfected with only Gag plasmid. 293T cells were co-transfected with Gag, pCEP4-Tat, and Env plasmid. Because these HIV-VLPs express EYFP, fluorescent units (FU) were used as an indicator of quantity. ELISA was also used to determine the concentrations of Gag protein in individual HIV-VLP batches using a p24 Ag capture assay. Ratios of gp120 to EYFP for a given batch were calculated to ensure similar quantities were administered per dose. An HIV-VLP dose of 300 FU equivalents conferred reliable stimulation and promoted peripheral immune cell infiltration into the brain.

Intracranial injection of mice

Injection of mice was performed as previously described with slight modifications (Cheeran et al., 2004). Briefly, female mice (6–8 week old) were anesthetized using a combination of Ketamine (Akorn, Inc. Lake Forest, IL) and Xylazine (Bimeda Inc., Le Sueur, MN), (100 mg and 10 mg/kg body weight, respectively) and immobilized on a small animal stereotactic instrument equipped with a Cunningham mouse adapter (Stoelting Co., Wood Dale, IL). Subcutaneous injection of the analgesic bupivacaine (Hospira, Inc. Lake Forest, IL), (1-2 mg/kg [0.4-0.8 ml/kg of a 0.25% solution]) in the area prior to incision was performed to minimize pain. The skin and underlying connective tissue were reflected to expose reference sutures (sagittal and coronal) on the skull. The sagittal plane was adjusted such that bregma and lambda were positioned at the same coordinates on the vertical plane. A burr hole was drilled to expose the underlying dura at pre-determined coordinates to access the left striatum (AP = 0 mm, ML = 2.0 mm from bregma, and DV = 3.0 from skull surface). Animals received 300 FU of HIV-VLP in a volume no greater than 5 µl delivered to striatum via stereotaxic injection, using a Hamilton syringe (10 µl) fitted to a 27 G

needle. The injection was delivered over a period of 5 min. The needle was retracted slowly and burr hole was closed with sterile bone-wax, the animal was removed from the stereotaxic apparatus and the skin incision was closed with 4-0 silk sutures with a FS-2 needle (Ethicon, Somerville NJ).

Ag Restimulation

Restimulation with HIV-1-specific T-cell epitope peptide was performed 30 d post HIV-VLP. Previous studies have identified the capsid p24 H-2K^d MHC class I-restricted peptide AMQMLKETI (i.e., AI9) as an immunodominant epitope (Tan et al., 2016; Tan et al., 2018). Animals were injected with 100 µM AI9 peptide in 5-µl saline delivered into the brain striatum. Brain tissue was isolated at 2 and 5 d post-restimulation. BMNC were examined for Ag-specific CD8$^+$ T-cells, as well as microglial cell activation, by flow cytometry, whereas brain tissue sections were collected at 2 d for IHC staining and qPCR.

Brain leukocyte isolation and flow cytometry analysis

BMNC were isolated from the brain of prime-CNS boost animals using a previously described procedure with minor modifications (Cheeran et al., 2007; Ford et al., 1995; Marten et al., 2003; Mutnal et al., 2011). In brief, whole brain tissues were harvested (n = 4-6 animals/group/experiment), and minced finely using a scalpel in RPMI 1640 (2 g/L D-glucose and 10 mM HEPES) and digested in 0.0625% trypsin (in Ca/Mg-free HBSS) at room temperature for 20 min. Single cell preparations of infected brains were resuspended in 30% Percoll (Sigma-Aldrich, St. Louis, MO) and banded on a 70% Percoll cushion at 900 × g for 30 min at 15°C. Brain leukocytes obtained from the 30–70% Percoll interface were collected. Following preparation of single cell suspensions, cells were treated with Fc block (anti-CD32/CD16 in the form of 2.4G2 hybridoma culture supernatant with 2% normal rat and 2% normal mouse serum) to inhibit nonspecific Ab binding. Cells were then counted using the trypan blue dye exclusion method, and

1 x 10^6 cells were subsequently stained with anti-mouse immune cell surface markers for 15-20 min at 4°C (anti-CD45-BV605, anti-KLRG1-PE-Cy7, anti-CD11b-AF700, anti-CD103-FITC, anti-CD127-PECy-5, anti-CD69-e-F 450, anti-CD49a-PE, anti-PD1-BV711 (Previously eBioscience, Thermo Fisher Scientific, Waltham, MA) and anti-CD8-BV-510 (clone YTS156.7.7) from (Biolegend, San Diego, CA). Control isotype Abs were used for all fluorochrome combinations to assess nonspecific Ab binding. For tetramer staining, the H-2K^d major histocompatibility complex (MHC) class I-restricted peptide AMQMLKETI (AI9) as the immunodominant T-cell epitope was purchased from MBL Corporation (Woburn, MA) and used for evaluation of Ag-specific responses. 200,000 events were recorded and live leukocytes were gated using forward scatter and side scatter parameters on a LSRII H4760 (BD Biosciences, San Jose CA). The gating strategy is shown in Supplementary Figures (S1B, C and D). Data were analyzed using FlowJo software (FlowJo, Ashland, OR).

Intracellular cytokine staining

To assess intracellular cytokine production, BMNC (2 x 10^6 cells/well) were incubated either with polyclonal stimulation using anti-CD3/CD28 or HIV-1-specific peptide for 5h at 37°C in RPMI complete medium supplemented with 10% FBS. Brefeldin A (1 µl/ml) was present throughout the incubation. Peptide was omitted in negative control samples. Cells were surface stained prior to fixation/permeabilization using cytofix/cytosperm kit (Thermo Fisher Scientific). Cells were then stained for Ki67-PE, IFN-γ, eF450, IL-2-PE-cy7, TNF-α-APC (Thermo Fisher Scientific) and Granzyme B-APC (Biolegend). Transcription factors associated with T_{RM} development like Blimp-1(Biolegend), Eomes (Invitrogen, Carlsbad, CA), and T-bet (Thermo Fisher Scientific) were stained, as recommended by manufacturer's protocol. Stained cells were analyzed as described above.

Real-time RT-PCR

Cultures of BMNC with or without $CD8^+$ T-cells (depleted through positive isolation using a Miltenyi Biotech Kit (Bergisch Gladbach, Germany) from post prime-CNS boost animals at 30 d were used. BMNC were either stimulated with AI9 peptide or left unstimulated for 1h before being co-cultured with mixed glial cells (40% microglia; 60% astrocytes) for 24 h. $CD8^+$ T-cells were added at 10:1 CD8: glial cell ratio. RNA was extracted from co-cultured cells using the RNeasy® Lipid Tissue Mini kit (Qiagen, Valencia, CA), treated with DNase and reverse transcribed to cDNA with oligo $(dT)_{12–18}$, random hexmer, dNTPs (Gene Link, Hawthorne, NY), RNase inhibitor and SuperScript™ III reverse transcriptase (Invitrogen). Diluted cDNA, primers and SYBR® Advantage® qPCR premix (ClonTech, now Takara Bio USA, Mountain View, CA) were subjected to real-time PCR (Bio-Rad Laboratories, Hercules, CA) according to the manufacturer's protocol. Primer sequences were: sense 5'-GCGTCATTGAATCACACCTG-3' and antisense 5'- GACCTGTGGGTTGTTGACCT-3' for IFN-γ (104 bp); sense 5'- GACGCTCAACTTGTCCCAAAAC -3' and antisense 5'- GCAGCCGTGAACTTGTTGAAC -3' for MHC-II (200 bp); sense 5'- CGTGAGTGGGAAGAGAAGTGTC-3' and antisense 5'- CTACAATGAGGAACAACAGGATGG-3' for PD-L1 (239 bp); sense 5'GACTTCCACATGAACATCCTTGAC-3' and antisense 5'CTCTGGCCTCTGACATACTTGTTG-3' for PD-1 (295 bp); sense 5'- GTCATTTTCTGCCTCATCCTGCT-3' and antisense 5'- GGATTCAGACATCTCTGCTCATCA-3' for CXCL10 (212 bp); sense 5'- AGAACGGAGATCAAACCTGCCT-3' and antisense 5'- CGACTTTGGGGTGTTTTGGGTT-3' for CXCL9 (157 bp); sense 5'- GGAGGGGATCAACAAGCAATTCCT-3' and antisense 5'- GGAGCCACTGGACACCTCTCTAAT-3' for Iba-1 (220 bp) sense 5'-

TGGCCACCTTGTTCAGCTACG-3’ and antisense 5’- GCCAAGGCCAAACACAGCATA-3’ for iNOS (212 bp); and sense 5’- TGCTCGAGATGTCATGAAGG-3’ and antisense 5’-AATCCAGCAGGTCAGCAAAG-3’ for HPRT (hypoxanthine phosphoribosyltransferase, 95 bp). The PCR conditions for the Bio-Rad CFX96 qPCR System were: 1 denaturation cycle at 95°C for 10 s; 40 amplification cycles of 95°C for 10 s, 60°C annealing for 10 s, and elongation at 72°C for 10 s; followed by 1 dissociation cycle. The relative product levels were quantified using the $2^{-\Delta\Delta Ct}$ method (Livak and Schmittgen, 2001) and were normalized to the housekeeping gene HPRT.

Primary glial cell cultures

Cerebral cortical cells from 1 d old Balb/c mice were dissociated after a 30 min trypsinization (0.25 %) in HBSS and plated in 75 cm2 culture flasks in DMEM containing 6 % FBS, penicillin (100 U/ml), streptomycin (100 µg/ml), gentamicin (50 µg/ml), and Fungizone® amphotericin B (250 pg/ml). The medium was replenished 1 and 4 d after plating. On 12 d of culture, floating microglial cells were harvested and plated onto 48-well cell culture plates (1×10^5 cells/well). After a 1 h incubation at 37 °C, the culture plates were washed and incubated overnight before starting the experiments. Purified microglial cell cultures were comprised of a cell population in which >95 % stained positively with Iba-1 antibodies and 3–5 % stained positively with antibodies specific to GFAP. Purified astrocyte cultures were prepared from the culture flask following isolation of microglia at 14 days *in vitro*. Briefly, after collection of microglia, the culture flasks were shaken at 180–200 rpm at 37 °C for 16 h followed by trypsinization (0.25 % trypsin in HBSS) for 30 min. Cells were seeded into new flasks with DMEM, after adding FBS (final concentration 10 %), centrifugation, and washing. The medium was changed after 24 h. This subculture procedure was repeated weekly for 2–3 times to remove residual oligodendrocytes and microglia,

in order to achieve highly purified astrocyte cultures (95–98 % of cells reacted with GFAP Ab, 3-5 % stained with Iba-1 Ab), which were plated onto 48-well culture plates (1×10^5 cells/well).

ELISA

The supernatants from AI9-stimulated BMNC (30 d post-prime-CNS boost) co-cultured either with purified microglial cells or astrocytes (48 h) were collected for ELISA. In brief, 96-well ELISA plates were pre-coated with anti-mouse CXCL9, CXCL10 (R&D Minneapolis, MN), or IFN-γ (Invitrogen) Abs (2 μg/ml) overnight at 4 °C and blocked with 1 % BSA in PBS for 1 h at room temperature (RT). After washing (PBS with Tween 20), supernatants and a series of diluted standards were added to the wells for 2 h at RT. Abs to anti-CXCL9, CXCL10 and IFN-γ were added and incubated for 90 min at RT followed by addition of secondary Abs conjugated with horseradish peroxidase (according to manufacturer's guidelines) for 45 min at RT. The chromogen substrate K-Blue (Neogen, Lexington, KY) was added for color development, which was terminated with 1 M H_2SO_4. The plates were read at 450 nm, and concentration levels of cytokine and chemokines were extrapolated from standard curves, normalized to protein concentrations.

Immunohistochemistry

Brains were harvested from prime-CNS boost mice with or without AI9 restimulation for 2 d. Animals were perfused with serial washes of 2% sodium nitrate and phosphate-buffered saline (PBS) to remove contaminating blood cells, and prefixed with 4% paraformaldehyde. Murine brains were subsequently submerged in 4% paraformaldehyde for 24 h and transferred to 25% sucrose solution for 2 d prior to sectioning. After blocking (PBS with 10% normal donkey serum and 0.3% Triton X-100) for 1 h at RT, brain sections (25 μm) were incubated overnight at 4°C with the following primary antibodies: Rat anti-mouse MHC-II (10 μg/mL; eBioscience, San Diego CA), Goat anti-mouse CXCL10 (10μg/mL; R&D Systems, Minneapolis, MN) , Rabbit anti

GFAP (1:500; DAKO, Sunnyvale, CA), Rabbit anti Iba-1 (2ug/ml; Wako chemicals, Richmond, VA) and Rabbit anti-TMEM119 antibody (Abcam, Cambridge, MA). Brain sections were washed four times with PBS. After washing, secondary antibody (Donkey anti Rat FITC-conjugate, Donkey anti Goat NL577-conjugate and Donkey anti NL493-conjugate was added for 1h at RT followed by nuclear labeling with Hoechst 33342 (1 μg/ml; Chemicon, Temecula, CA) and viewed under a fluorescent microscope.

Statistical analysis

For comparing groups, a two-tailed unpaired Student's T-test for samples was applied, *p* values ≤ 0.05 were considered significant.

Supplemental information

Figure S1: Expression of HIV-p24 in murine liver (related to Figure 1). A. IHC staining of murine liver sections for HIV-1 p24 protein 7 d post-priming via intravenous injection with rAd5-p24 (1 x 10^{10} PFU) confirms that the construct delivers bona fide p24 protein which is expressed *in vivo*. **B.** Flow cytometry gating strategy for the rAd5-p24/Sal group. **C.** Flow cytometry gating strategy for the rAd5-p24/ HIV-VLP group. **D.** Quantification of tetramer signaling on non-$CD8^+$ T-cells at 7 d and 30 d post-prime CNS boost**.**

Figure S2: Frequency of $CD4^+$ and $CD8^+$ T-cells (related to Figure 1 & Figure 4). A. Frequency of $CD4^+$ and $CD8^+$ T-cells at the indicated time points among rAd5-p24/ HIV-VLP animals. **B.** Representative plot showing successful depletion of $CD8^+$ T-cells isolated from the

brain of post prime-CNS boost animals using the Miltenyi Biotech Kit (Bergisch Gladbach Germany).

Figure S3: Gating strategy for microglial cells (related to Figure 6). A. Microglia were identified as the $CD45^{int}CD11b^{+}$ population within total BMNC. **B.** Representative images of microglial cell populations showing expression of the activation marker MHC-II in respective control groups. **C.** Contour plots show expression of the microglial activation marker PD-L1 in the indicated groups (i.e., treatment with either rAd5-p24, HIV-VLP, or rAd5-p24/Sal).

Figure S4: Cytotoxic potential of $CD103^{+}CD8^{+}$ bT_{RM} following AI9 restimulation (related to Figure 1 & Figure 6). BMNC were obtained from heterologous prime-boost animals at 2 & 5 d post-AI9 restimulation *in vivo* and intracellular staining was performed. **A.** Representative contour plots display granzyme B production by $CD103^{+}CD8^{+}$ T-cells in response to AI9 peptide, control M54 peptide, or saline at the indicated time points. **B.** Bar graph indicates frequencies of $CD103^{+}CD8^{+}$ T-cells that produce granzyme B under the indicated restimulation conditions. Pooled data are presented as mean ± SD of two independent experiments using four animals in control (i.e., saline or M54) groups and six animals in the AI9 restimulation group. $^{**}p<0.01$

Figure S1

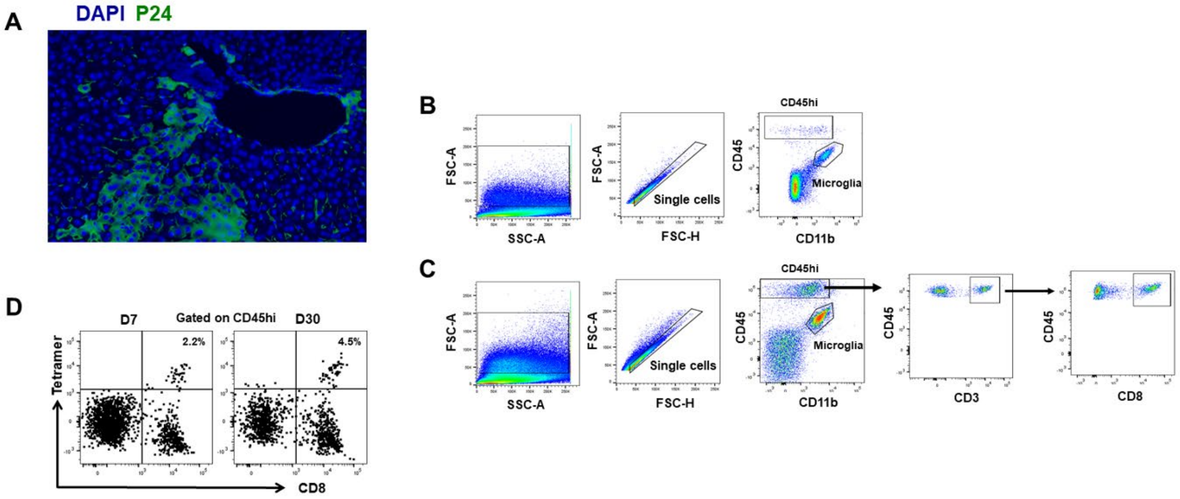

Figure S2

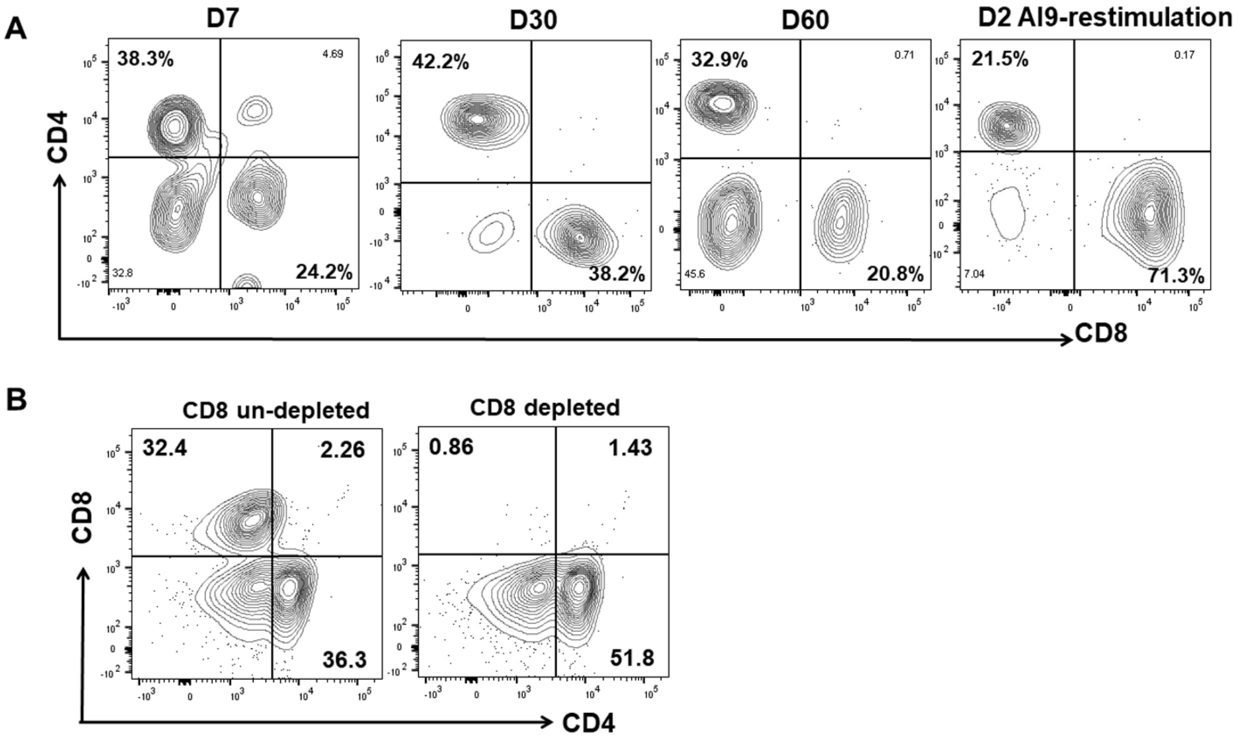

Figure S3

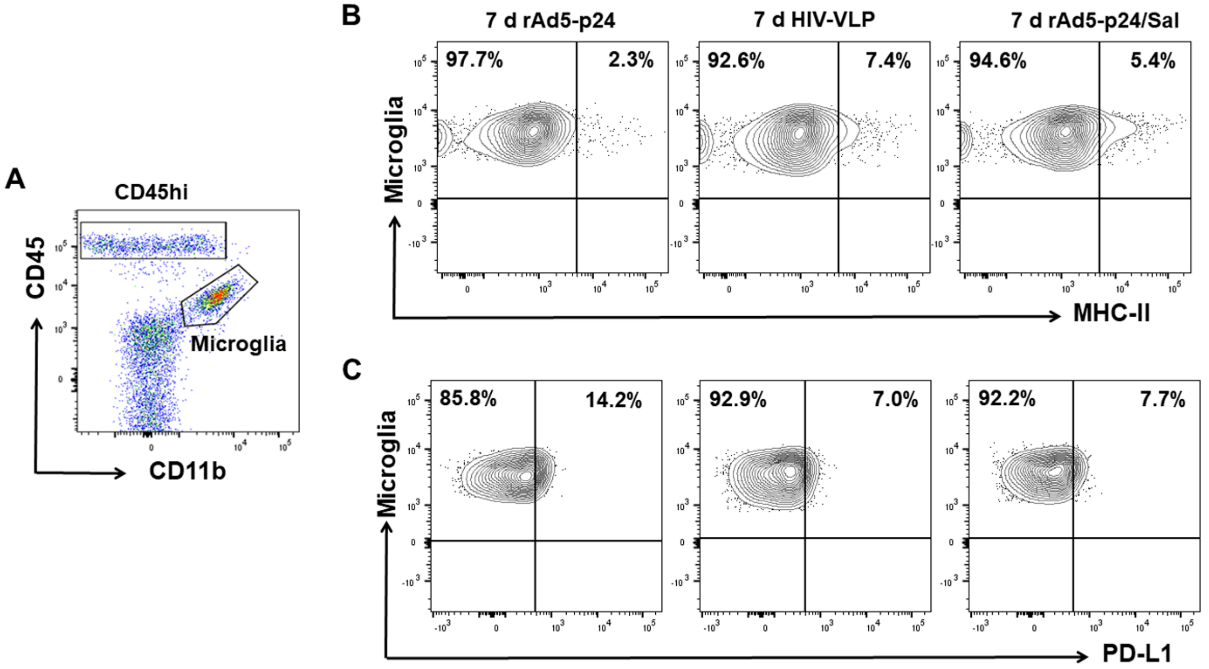

Figure S4

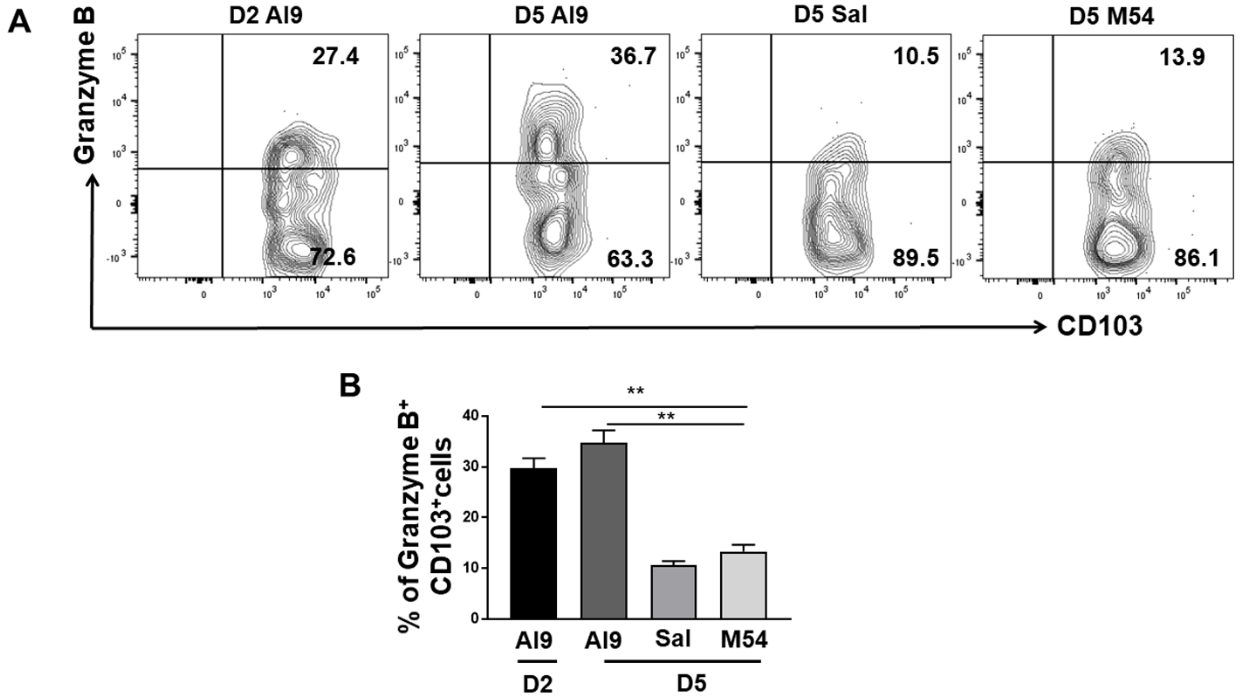

Supplement: Document S1. Transparent Methods and Figures S1–S4 [file mmc1.pdf]
